# An open access journal of molecular signaling: a critical need at a critical time

**DOI:** 10.1186/1750-2187-1-1

**Published:** 2006-11-10

**Authors:** Danny N Dhanasekaran

**Affiliations:** 1 Fels Institute for Cancer Research and Molecular Biology, Temple University School of Medicine, 3307 N. Broad Street, Philadelphia, PA 19140, USA

## Abstract

Molecular signaling is an exponentially growing field that encompasses different molecular aspects of cell signaling underlying normal and pathological conditions. This area also focuses on defining the genetic and epigenetic changes that modulate the signaling properties of cells and the resultant physiological as well as pathological conditions. Therefore, rapid publication of results from these endeavors and, more importantly, free access to such publications can truly accelerate the progress in this field leading to the development of novel targeted drugs. With this goal in mind, Journal of Molecular Signaling, a journal fully devoted to open access publishing of rigorously peer-reviewed quality manuscripts in the molecular signaling area of research, is being launched. The focus, significance, and, the open access model of publishing of Journal of Molecular Signaling are discussed.

## Editorial

### An open access journal of molecular signaling: a critical need at a critical time

*"The free, unhampered exchange of ideas and scientific conclusions is necessary for the sound development of science, as it is in all spheres of life." (Albert Einstein, 1952)*.

At a time where new theories and discoveries are being made almost on a daily basis, it is essential for the scientific community to have free and ready access to these new findings in order to accelerate further progress. It is also important to make sure that the dissemination of scientific information transcends the geopolitical as well as economical barriers so that it reaches all in the far corners of the world. A forum for such a free and rapid flow of scientific information is acutely needed for the molecular signaling area of research. Molecular signaling is an exponentially growing field that encompasses different molecular aspects of cell signaling underlying normal and pathological conditions. This area also focuses on defining the genetic and epigenetic changes that modulate the signaling properties of cells and the resultant physiological as well as pathological conditions. Defining molecular events in cell signaling forms the basis for identifying novel diagnostic, prognostic, and therapeutic targets for many different diseases. Therefore, rapid publication of results from these endeavors and, more importantly, free access to such publications can truly accelerate the progress in this field leading to the development of novel targeted drugs. With this goal in mind, *Journal of Molecular Signaling *under the umbrella of BioMed Central's open access publishing platform, will be fully devoted to open access publishing of rigorously peer-reviewed quality manuscripts in the molecular signaling area of research.

The open access model of publishing has several advantages over the traditional subscription-based access (both print as well as online) to research information [1,2]. The immediate benefits to the authors are 1) the free and universal visibility of the publication 2) author retention of copyright privileges for the work along with the license to allow anyone to reproduce and disseminate the published article provided due attribution is given to the author and 3) the archival of the published articles in the repositories such as PubMed Central of the US National Library of Medicine [3], University of Potsdam in Germany [4], INIST in France [5], and e-depot of National Library of the Netherlands [6]. Likewise, the key benefits of open access to the scientific community and the general public can also be enumerated [7-12]. First, open access allows authors to reach a large number of targeted readers immediately and irrespective of their locations [7,11,12]. In fact, such an enhanced readership in the open access model has been correlated with the progressively increasing impact factor of these journals [8-10]. Second, open access readily eliminates the economical disparity in accessing vital scientific information [11,12]. Third, the central archiving that has become inherent to the open access journals, allows enhanced literature search and meta-analyses of data [3-6,13]. Fourth, considering the fact that most research endeavors are funded by the tax-payers, open access journals strive to make sure that the outcome of these research efforts are accessible to the public free of cost [14-17].

The major focus of *Journal of Molecular Signaling *will be on the signaling pathways involved in proliferation, differentiation, apoptosis, and oncogenesis in the mammalian systems. Specifically, the research area of our journal is on the normal or aberrant molecular mechanisms involving receptors, G-proteins, kinases, phosphatases, and transcription factors in regulating cell proliferation, differentiation, apoptosis, and oncogenesis in mammalian cells. With the launch of this journal, we hope to provide up-to-date information to researchers in this rapidly growing field of interest that is molecular signaling. With this journal, our goals are to a) simplify the search process by focusing on a specific set of signaling pathways and b) provide a high quality scientific journal in an open access forum at the touch of a keyboard. We have an excellent editorial board consisting of highly distinguished scientists [18], who will assure fair and unbiased peer-review. Articles will be published immediately upon acceptance and the published articles will be indexed in PubMed [19] and archived in the repositories previously noted, including PubMed Central [3-6].

We, the editorial team of the *Journal of Molecular Signaling*, will make every effort to publish exceptional and interesting manuscripts that will have great impact in molecular and cell signaling research. We also hope to publish topical reviews and commentaries from the experts in different areas of signaling research. With your comments and input, we will ensure that the journal meets your needs. We are looking forward to the submission of your next important manuscript to the *Journal of Molecular Signaling *for the continued success of this journal.
